# The *In Vivo* Role of the RP-Mdm2-p53 Pathway in Signaling Oncogenic Stress Induced by pRb Inactivation and Ras Overexpression

**DOI:** 10.1371/journal.pone.0021625

**Published:** 2011-06-29

**Authors:** Wenqi Pan, Sameer Issaq, Yanping Zhang

**Affiliations:** 1 Department of Radiation Oncology, University of North Carolina at Chapel Hill, Chapel Hill, North Carolina, United States of America; 2 Lineberger Comprehensive Cancer Center, University of North Carolina at Chapel Hill, Chapel Hill, North Carolina, United States of America; 3 Department of Pharmacology, University of North Carolina at Chapel Hill, Chapel Hill, North Carolina, United States of America; 4 Curriculum in Genetics and Molecular Biology, School of Medicine, University of North Carolina at Chapel Hill, Chapel Hill, North Carolina, United States of America; Wayne State University School of Medicine, United States of America

## Abstract

The Mdm2-p53 tumor suppression pathway plays a vital role in regulating cellular homeostasis by integrating a variety of stressors and eliciting effects on cell growth and proliferation. Recent studies have demonstrated an *in vivo* signaling pathway mediated by ribosomal protein (RP)-Mdm2 interaction that responds to ribosome biogenesis stress and evokes a protective p53 reaction. It has been shown that mice harboring a Cys-to-Phe mutation in the zinc finger of Mdm2 that specifically disrupts RP L11-Mdm2 binding are prone to accelerated lymphomagenesis in an oncogenic c-Myc driven mouse model of Burkitt's lymphoma. Because most oncogenes when upregulated simultaneously promote both cellular growth and proliferation, it therefore stands to reason that the RP-Mdm2-p53 pathway might also be essential in response to oncogenes other than c-Myc. Using genetically engineered mice, we now show that disruption of the RP-Mdm2-p53 pathway by an Mdm2^C305F^ mutation does not accelerate prostatic tumorigenesis induced by inactivation of the pRb family proteins (pRb/p107/p130). In contrast, loss of p19Arf greatly accelerates the progression of prostate cancer induced by inhibition of pRb family proteins. Moreover, using ectopically expressed oncogenic H-Ras we demonstrate that p53 response remains intact in the Mdm2^C305F^ mutant MEF cells. Thus, unlike the p19Arf-Mdm2-p53 pathway, which is considered a general oncogenic response pathway, the RP-Mdm2-p53 pathway appears to specifically suppress tumorigenesis induced by oncogenic c-Myc.

## Introduction

p53 is a critical tumor suppressor gene which is mutated in about 50%of all human tumors [1]. It is often referred to as the guardian of the genome because under various cellular stress conditions such as DNA damage, oncogenic insult, and hypoxia, p53 is stabilized and activated, inducing cell cycle arrest, apoptosis, DNA damage repair, senescence, and a variety of other protective responses [2]. Under normal conditions, p53 levels are kept low, mainly through inhibition by Mdm2 (mouse double minute 2). The C-terminus of Mdm2 has an intrinsic E3 ligase activity, which promotes the ubiquination and degradation of p53. The N-terminus of Mdm2 binds to the transactivation domain of p53 and inhibits the recruitment of co-activators. Mdm2 is also directly transactivated by p53, therefore forming an Mdm2-p53 feedback loop to maintain cellular homeostasis [3].

Recently several ribosomal proteins, including L11 [4], L5 [5] and L23 [6], [7] have been shown to bind Mdm2 at its zinc finger domain. Under normal conditions, these proteins, along with rRNAs, form the large and small subunits of ribosomes in the nucleolus [8]. However, under conditions of ribosome stress, free forms of ribosomal proteins are released into the nucleoplasm and bind to Mdm2, leading to p53 stabilization and activation [9]. A cancer-associated cysteine-to-phenylalanine point mutation in the zinc finger domain of Mdm2 causes disruption of L11 and L5 binding to Mdm2 [10], and based on this *in vitro* data, we previously generated a knock-in mouse with the Mdm2 C305F mutation. Mdm2^C305F^ mutant mice maintain a normal p53 response to DNA damage, but are deficient in p53 induction in response to induced ribosomal stress [11].

Intriguingly, the Mdm2 C305F mutation was recently shown to significantly accelerate B cell lymphomagenesis in an Eµ-Myc induced mouse model of B cell lymphoma [11]. The ability of Myc to promote cell growth and proliferation is closely linked to its role in regulating ribosomal biogenesis. Myc facilitates the recruitment of Pol I to rDNA promoters [12], [13], promotes the transcription of ribosomal proteins by activating Pol II [14], [15], [16], [17], and activates Pol III-mediated transcription of 5S rRNA and tRNA [18]. In the case of Eµ-myc-induced lymphoma, ribosomal proteins L11 and L5 are unable to bind and suppress Mdm2^C305F^ in Eµ-*Myc;Mdm2^C305F/C305F^* mice, and as a result activation of p53 is attenuated and B cell lymphomagenesis is accelerated [11]. These findings established the RP-Mdm2-p53 pathway as a genuine barrier to Myc-induced tumorigenesis.

Another well-studied pathway suppressing Myc-induced B cell lymphoma is ARF-Mdm2-p53 signaling. Loss of p19Arf results in a similar acceleration of Eµ-Myc induced lymphomagenesis to that caused by Mdm2 C305F mutation [11], [19]. ARF can physically interact with and inhibit Mdm2, therefore releasing p53 from Mdm2-mediated degradation and transactivation silencing [20], [21], [22], [23]. Besides Myc, ARF can also induce p53 in response to E2F1 and Ras. E2F1 directly activates human p14ARF at the transcriptional level [24]. Overexpression of Ras transforms p19Arf-null mouse embryo fibroblasts (MEFs) via bypassing p53-mediated checkpoint control [25]. Ras also induces cell cycle arrest in wild-type murine keratinocytes, which is mediated by increased expression of p19Arf [26]. While ARF-Mdm2-p53 signaling acts downstream of a variety of oncogenes, ARF-independent induction of p53 can also occur upon oncogenic stress. For instance, when expressing T121, a transgene inhibiting pRb and therefore activating E2F1, in choroid plexus (CP) epithelial cells, p19Arf is dispensable for p53-mediated tumor suppression and apoptosis [27]. Ras induction of p53-dependent cell cycle arrest in murine keratinocytes also does not rely on ARF [28]. The alternative pathway leading to p53 activation is unclear. Given that oncogenes promote cell proliferation and/or growth associated with elevated protein synthesis, ribosomal biogenesis might be generally disrupted in response to oncogenic stress. Therefore, RP-Mdm2-p53 signaling may play a general role in responding to oncogenic stress and suppressing tumorigenesis like it does in Myc-induced B cell lymphoma.

E2F1 has been reported to bind the promoters of rRNA and enhance its activity [29]. Similarly, in the yeast *Saccharomyces cerevisiae*, Ras/TOR induces Sfp1 (zinc finger-containing transcription factor), which activates RP gene expression, a network linking cell growth to ribosomal biogenesis [30]. In mammalian cells, Ras-PI3K-Akt-mTOR signaling pathway is well-known to promote protein translation and cell growth [31]. Upregulation of these cellular processes may induce ribosomal stress, leading to activation of RP-Mdm2-p53 signaling. Hence, the current study focuses on examining whether the RP-Mdm2-p53 pathway may act as a general response to oncogenic stress by utilizing models of pRb inactivation and Ras activation.

Specifically, to investigate whether disruption of RP-Mdm2-p53 signaling by Mdm2^C305F^ mutation accelerates tumorigenesis induced by inactivation of pRb, we crossed *Mdm2^C305F^* mice with the well-characterized *APT_121_* mouse prostate cancer model, in which a truncated SV40 large T antigen (consisting of the first 121 N-terminal amino acids; *T_121_*) controlled by the probasin promoter leads to pRb inactivation in prostate epithelium to induce prostate cancer [32], [33]. To investigate whether disruption of RP-Mdm2-p53 signaling accelerates tumorigenesis induced by Ras activation, we used mouse keratinocyte and mouse embryonic fibroblast systems to measure Ras-induced ribosomal protein levels and p53 response signaling.

## Results

### Mdm2 C305F mutation causes reduced prostate size and slows the progression of *APT_121_*-induced prostate cancer

Inactivation of p53 alone in the murine prostate leads to the development of prostatic intraepithelial neoplasia (PIN) with no progression to invasive carcinoma, suggesting that loss of p53 may be a complementary rather than initiating event in promoting prostate tumorigenesis [34]. Previous findings have also shown that attenuation of p53 signaling through loss of one allele of p53 does not accelerate the onset of epithelial tumors in an *APT_121_*-induced mouse model of prostate cancer, but induces a stromal tumor phenotype, which is characterized by extensive stromal cell presence and intraductal growth patterns [35]. The Mdm2 C305F mutation, which disrupts the binding of ribosomal proteins L11 and L5 to Mdm2 [11], causes an attenuation of p53 signaling, suggesting that the Mdm2 C305F mutation may alter the progression, rather than initiation, of prostate tumorigenesis in a similar way as p53 heterozygosity.

To examine the importance of the RP-Mdm2-p53 pathway in *APT_121_*-induced prostate cancer, we generated *APT_121_;Mdm2^+/+^* and *APT_121_;Mdm2^C305F/C305F^* mice and non-tumorigenic control *Mdm2^+/+^* and *Mdm2^C305F/C305F^* mice. The progression of tumorigenesis was then compared among these mice to see if disruption of RP-Mdm2-p53 signaling altered the development of cancer.

**Figure 1:**
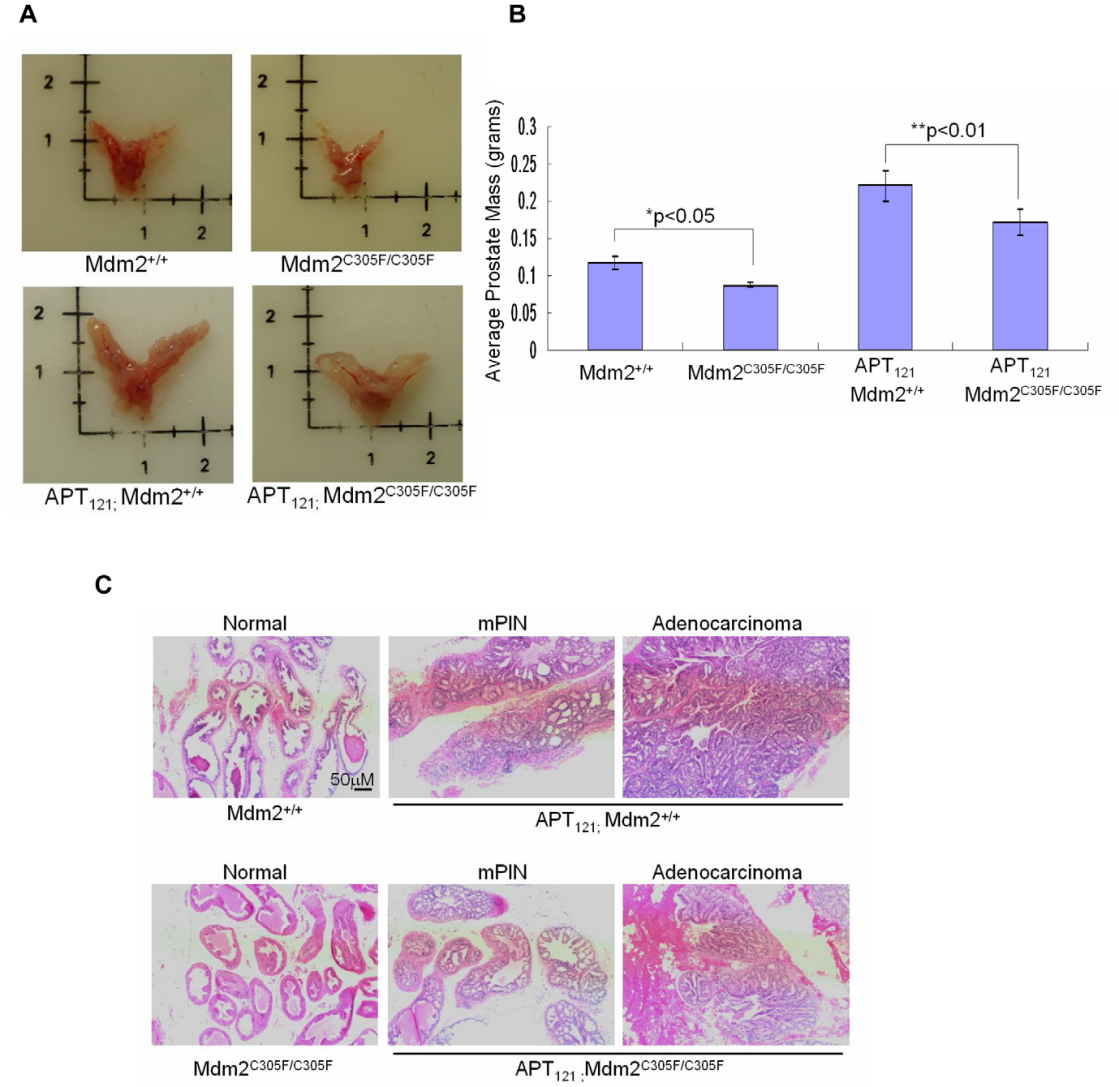
Mdm2 C305F mutation causes reduced prostate size and slows the progression of *APT_121_*-induced prostate cancer. A. Photographs showing representative prostates from 6 month-old mice of the indicated genotypes. B. Average prostate mass ± SD from 6 month-old mice of the indicated genotypes. *Mdm2^+/+^* (n = 12), *Mdm2^C305F/C305F^* (n = 11), *APT_121_;Mdm2^+/+^* (n = 9), and *APT_121_;Mdm2^C305F/C305F^* (n = 13) . * p<0.05 and ** p<0.01 as assessed by Student's t test. C. Representative H&E staining of prostate sections from 6 month-old mice of the indicated genotypes demonstrating histology associated with the indicated stages of tumor progression. Scale bar was shown in the first picture and all pictures were taken at the same magnification.

*APT_121_;Mdm2^+/+^* and *APT_121_;Mdm2^C305F/C305F^* mice did not exhibit noticeable differences in general appearance or body weight. We compared the size of prostate glands isolated from mice at 6 months of age. Surprisingly, the prostates from *Mdm2^C305F/C305F^* mice were generally smaller than those from *Mdm2^+/+^* mice, and consistent with this finding, the prostates from *APT_121_;Mdm2^C305F/C305F^* mice were smaller than those from *APT_121_;Mdm2^+/+^* mice (Figure 1A). The average weight of 11 *Mdm2^C305F/C305F^* prostates was 0.088 grams while that of 12 *Mdm2^+/+^* prostates was 0.117 grams. The average weight of 13 *APT_121_;Mdm2^C305F/C305F^* prostates was 0.172 grams and that of 9 *APT_121_;Mdm2^+/+^* prostates was 0.221 grams (Figure 1B). The differences in weight were statistically significant, with *p<0.05 and **p<0.01 respectively.

We next examined prostate histology by hematoxylin and eosin (H&E) staining on paraffin-embedded prostate samples isolated from 6 month-old mice. None of the *Mdm2^C305F/C305F^* or *Mdm2^+/+^* mice exhibited abnormality in their prostates (Figure 1C). Prostate adenocarcinoma, defined as penetration of malignant prostate epithelial cells through the basement membrane of the prostate gland into the surrounding stroma, was often observed in *APT_121_;Mdm2^+/+^* mice, while the majority of the *APT_121_;Mdm2^C305F/C305F^* mice only developed mPIN (mouse prostatic intraepithelial neoplasia), with few examples of well-differentiated adenocarcinoma (Figure 1C ). As shown in Table 1, 71.4%of *APT_121_;Mdm2^+/+^* mice developed adenocarcinomas compared with only 37.5%of *APT_121_;Mdm2^C305F/C305F^* mice. Thus the progression from mPIN to adenocarcinoma is decreased by Mdm2^C305F^ mutation.

**Table 1:** Summary of prostate tumor stages in 6 month-old *Mdm2^+/+^, Mdm2^C305F/C305F^*, *APT_121_;Mdm2^+/+^* , and *APT_121_; Mdm2^C305F/C305F^* mice.

	Mdm2^+/+^	Mdm2^C305F/C305F^	APT_121_;Mdm2^+/+^	APT_121_; Mdm2^C305F/C305F^
Total	8	7	7	8
Normal	8	7	0	0
Dysplasia	0	0	0	0
mPIN	0	0	2	5
Adenocarcinoma	0	0	5 (71.4%)	3 (37.5%)

### Mdm2 C305F mutation decreases proliferation but does not affect apoptosis of *APT_121_*-induced prostate cancer

**Figure 2:**
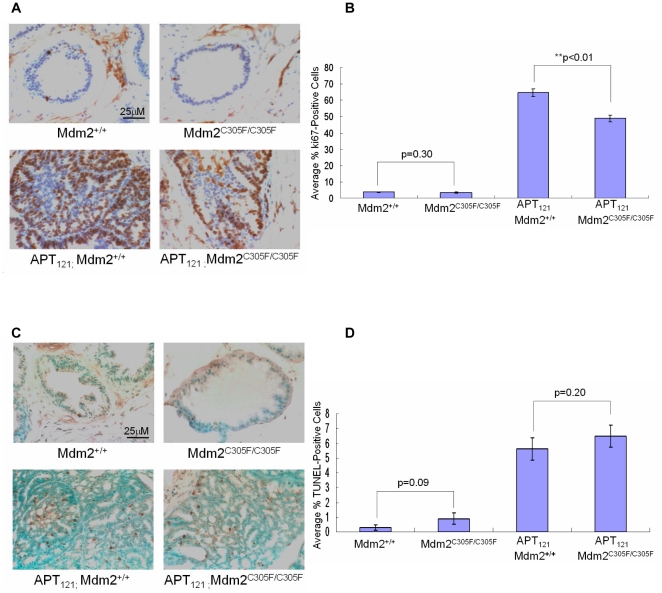
Mdm2 C305F mutation decreases proliferation but does not affect apoptosis of *APT_121_*-induced prostate cancer. A. Representative Ki67 staining of prostate sections from 6 month-old mice of the indicated genotypes. Brown staining indicates proliferating cells. Scale bar was shown in the first picture and all pictures were taken at the same magnification. B. Average%Ki67-positive cells ± SD from 6 month-old mice of the indicated genotypes. At least five independent fields consisting of a total of at least 1,000 cells from each prostate sample were counted. **p<0.01 as assessed by Student's t test. C. Representative TUNEL staining of prostate sections from 6 month-old mice of the indicated genotypes. Brown staining indicates apoptotic cells. Scale bar was shown in the first picture and all pictures were taken at the same magnification. D. Average%TUNEL-positive cells ± SD from 6 month-old mice of the indicated genotypes. At least five independent fields consisting of a total of at least 1,000 cells from each prostate sample were counted. (A–D) *Mdm2^+/+^* (n = 8), *Mdm2^C305F/C305F^* (n = 7), *APT_121_;Mdm2^+/+^* (n = 7), and *APT_121_;Mdm2^C305F/C305F^* (n = 9) mice were used.

To address the differences in tumor progression described above, the proliferation and apoptosis of isolated prostate tissues were examined by immunohistochemical analysis. Cell proliferation was assessed by ki67 staining. Prostates from *Mdm2^C305F/C305F^* or *Mdm2^+/+^* mice had few proliferating cells, while prostates from *APT_121_;Mdm2^+/+^* and *APT_121_;Mdm2^C305F/C305F^* mice were highly proliferative (Figure 2A). As quantified in Figure 2B, there was no statistically significant difference in the percentage of ki67 positive cells between *Mdm2^+/+^* and *Mdm2^C305F/C305F^* prostates (3.64%and 3.39%, respectively). However, there was a statistically significant difference in the percentage of ki67 positive cells between *APT_121_;Mdm2^+/+^* and *APT_121_;Mdm2^C305F/C305F^* prostates (64.6%and 48.8%, respectively).

To examine apoptosis in the prostates of the various transgenic mice, TUNEL (terminal deoxynucleotidyl transferase–mediated dUTP-biotin nick end labeling) immunohistochemical analysis was carried out. Representative pictures of TUNEL-stained sections are shown in Figure 2C. Prostates isolated from *APT_121_;Mdm2^+/+^* and *APT_121_;Mdm2^C305F/C305F^* mice had a much higher percentage of TUNEL-positive apoptotic cells than those of *Mdm2^+/+^* or *Mdm2^C305F/C305F^* mice (Figure 2D). However, there was no significant difference in apoptosis between *APT_121_;Mdm2^+/+^* and *APT_121_;Mdm2^C305F/C305F^* prostates (5.59%and 6.51%respectively) or between *Mdm2^+/+^* and *Mdm2^C305F/C305F^* prostates (0.29%and 0.89%respectively). Taken together, these data suggest that the Mdm2^C305F^ mutation may slow down the progression of prostate tumorigenesis by decreasing proliferation, rather than affecting the apoptosis of prostatic cells.

Previous studies have shown that Myc can up-regulate ribosomal biogenesis [12], [13] and that ribosomal protein expression is elevated during Myc-induced lymphomagenesis [11]. To investigate whether *APT_121_* induces increased expression of ribosomal proteins, total protein was isolated from prostate glands harvested from four mice of each genotype, and expression of ribosomal protein L11 was examined by western blot. Unlike the situation in Myc-induced lymphomagenesis in which L11 was significantly increased in the presence of Myc [11], L11 was not induced by *APT_121_* (data not shown), suggesting that *APT_121_*-induced prostate cancer does not cause ribosomal stress.

### Loss of p19Arf accelerates adenocarcinoma and stromal tumor development in *APT_121_*-induced prostate cancer

While our data suggest that RP-Mdm2 signaling does not inhibit *APT_121_*-induced prostate cancer, previous findings have shown that both RP-Mdm2 and p19Arf-Mdm2 signal to p53 and function equivalently as barriers to suppress Myc-induced B cell lymphoma [11], [19]. ARF can be induced by a variety of oncogenes including Ras, Myc and E2F1, inhibiting Mdm2 and thereby activating p53 [24], [36], [37]. p53 is believed to play an important role in suppressing prostate cancers of higher tumor stage or androgen-independent tumors [38], [39]. However, it is unknown whether p19Arf-Mdm2-p53 signaling is needed for the suppression of *APT_121_*-induced prostate cancer.

**Figure 3:**
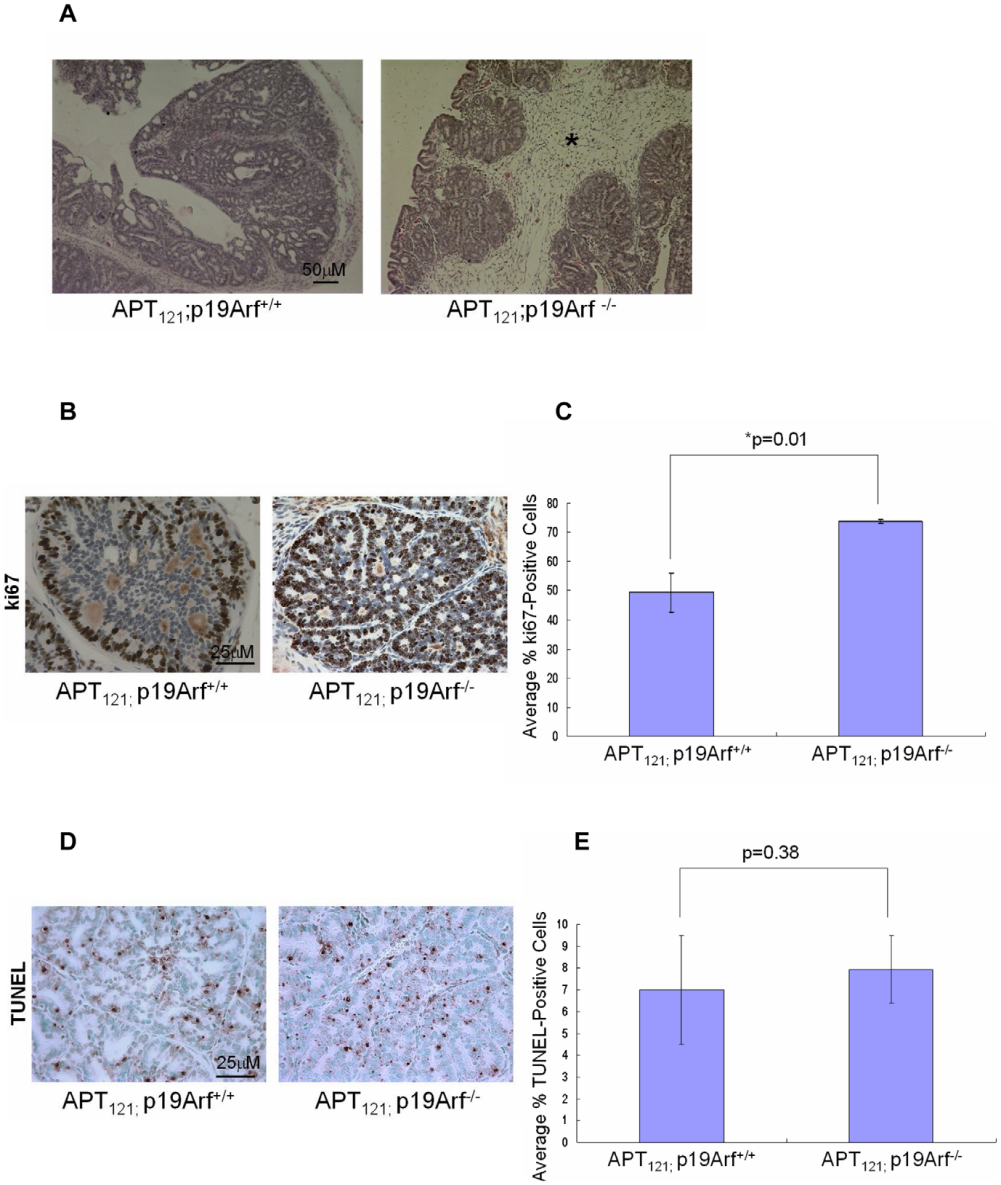
Effects of p19Arf loss on tumor progression in *APT_121_*-induced prostate cancer. A. Representative H&E staining of prostate sections from 5 month-old mice of the indicated genotypes. Stromal tumor was detected only in *APT_121_*;p19Arf^−/−^ mice as indicated by asterisk. Scale bar was shown in the first picture and all pictures were taken at the same magnification. B. Representative Ki67 staining from 5 month-old mice of the indicated genotypes. Scale bar was shown in the first picture and all pictures were taken at the same magnification. C. Average%Ki67-positive cells ± SD from 5 month-old mice of the indicated genotypes. n = 6 for each genotype. At least five independent fields consisting of a total of at least 1,000 cells from each prostate sample were counted. Brown staining indicates proliferating cells. *p = 0.01 as assessed by Student's t test. D. Representative TUNEL staining from 5 month-old mice of the indicated genotypes. Scale bar was shown in the first picture and all pictures were taken at the same magnification. E. Average%TUNEL-positive cells ± SD from 5 month-old mice of the indicated genotypes. n = 6 for each genotype. At least five independent fields consisting of a total of at least 1,000 cells from each prostate sample were counted. Brown staining indicates apoptotic cells.

To examine the role of the p19Arf-Mdm2-p53 pathway in *APT_121_*-induced prostate cancer, we crossed *APT_121_* with *p19Arf*-null mice. By 5 months of age, all *APT_121_*;*p19Arf^−/−^* mice developed adenocarcinomas, while the majority of the *APT_121_*;*p19Arf^+/+^* mice only developed mPIN (Table 2). Furthermore, tumors from *APT_121_*;*p19Arf^−/−^* prostates were comprised of a large portion of stromal cells, which expanded not only outside of the epithelial glands, but inside the glands as well (Figure 3A). This phenotype was similar to what was defined as ‘stromal tumor’ in a previous study [35]. The stromal tumor phenotype occurred at a high frequency (5 of 6 mice) in *APT_121_*;*p19Arf^−/−^* mice while it was not detected in *APT_121_*;*p19Arf^+/+^* mice (Table 2).

**Table 2:** Summary of prostate tumor stages in 5 month-old *APT_121_*;p19Arf^+/+^ and *APT_121_*;p19Arf^−/−^ mice.

		APT_121_;p19Arf^+/+^	APT_121_;p19Arf^−/−^
Total		6	6
Epithelial neoplasia	mPIN	4	0
	Adenocarcinoma	2 (33.3%)	6 (100%)
Stromal tumor		0	5 (83.3%)

We further measured the proliferation and apoptosis rates of *APT_121_;p19Arf^+/+^* and *APT_121_;p19Arf^−/−^* prostates by immunohistochemical analysis as mentioned above. *APT_121_;p19Arf^−/−^* prostates exhibited a higher rate of proliferation and no significant difference in apoptosis compared with *APT_121_;p19Arf^+/+^* prostates (Figure 3B–C and 3D–E). Therefore, p19Arf-Mdm2-p53 signaling apparently inhibits the progression of *APT_121_*-induced prostate cancer by affecting cell proliferation. Taken together, these data suggest that the p19Arf-Mdm2-p53 pathway, rather than the RP-Mdm2-p53 pathway, is the main barrier to suppress *APT_121_*-induced prostate cancer.

### Activated Ras does not up-regulate the expression of ribosomal protein in mouse keratinocytes

To further investigate if the RP-Mdm2-p53 signaling pathway is required for oncogenic Ras induction of p53, we examined its function in response to activation of H-Ras. Constitutively active mutant forms of the Ras family of small GTPases are found in approximately one-third of all human cancers. Active GTP-bound Ras stimulates numerous effector proteins to induce diverse downstream signaling events affecting cell growth, proliferation, differentiation, and apoptosis [40]. Given that the Ras-PI3K-Akt-mTOR pathway promotes protein translation and cell growth in mammalian cells [41], we tested whether activated Ras could induce ribosomal stress and trigger the RP-Mdm2-p53 pathway.

**Figure 4:**
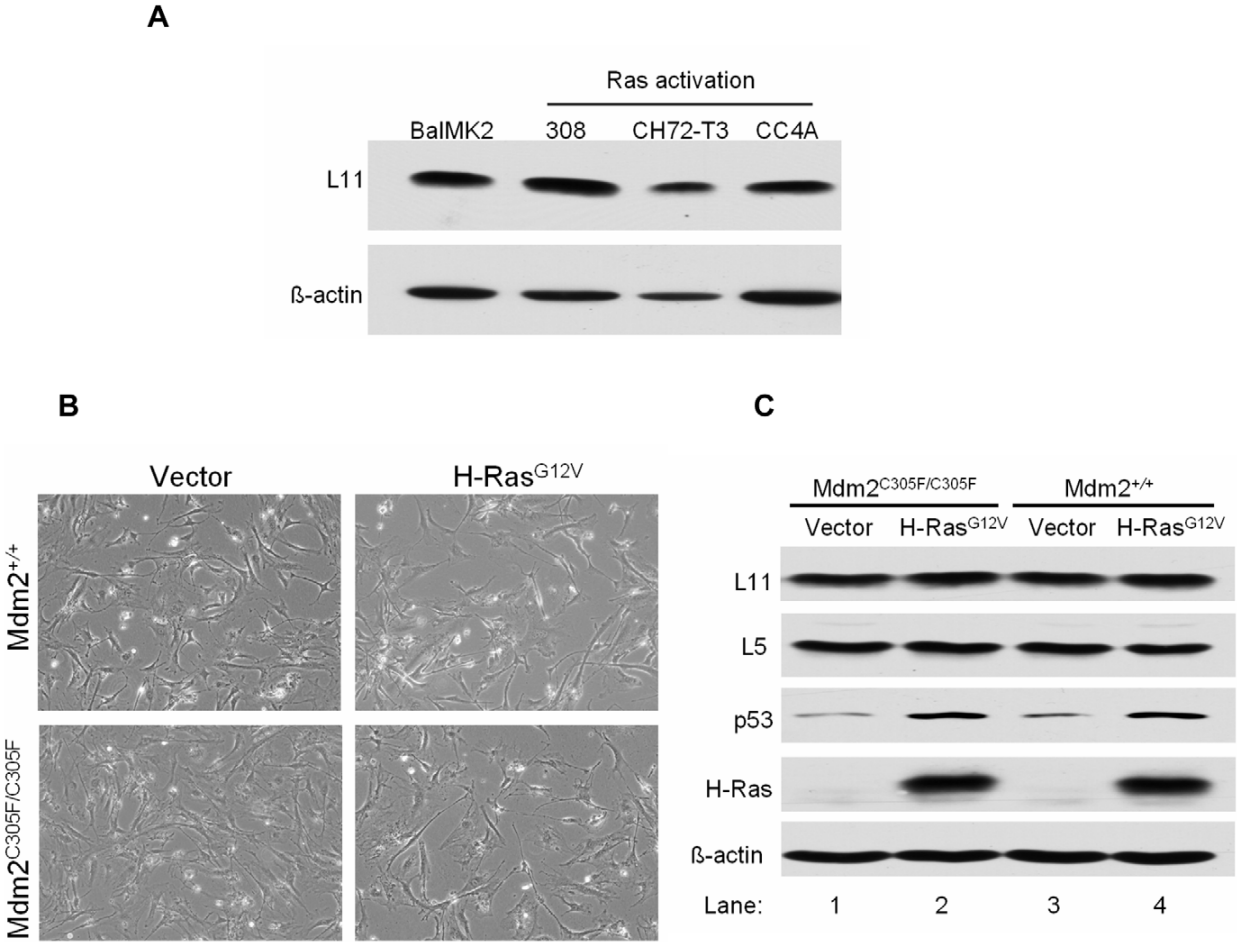
Activated Ras induces a normal p53 response but does not up-regulate ribosomal protein L11. A. Detection of L11 and β-actin by immunoblot analysis of total cellular lysate prepared from the indicated mouse karatinocyte cell lines. β-actin serves as a loading control. B. Representative phase-contrast images of *Mdm2^+/+^* and *Mdm2^C305F/C305F^* MEFs stably infected with empty vector or H-Ras^G12V^ retroviruses. C. Detection of L11, L5, p53, H-Ras, and β-actin by immunoblot analysis of total cellular lysate prepared from the MEFs described in B. β-Actin serves as a loading control.

To investigate this possibility, we first examined whether Ras could up-regulate the expression of ribosomal proteins in four different mouse keratinocyte cell lines: BalMk2 normal mouse keratinocytes with wild-type Ras, 308 benign mouse skin papilloma cells [42], CH72-T3 malignant mouse skin squamous cell carcinoma cells [43], and CC4A malignant mouse skin carcinoma cells all carrying an H-Ras mutation at codon 61 [44]. We measured the protein level of ribosomal protein L11 by western blot. Compared with BalMK2 normal mouse keratinocytes that have wild-type Ras, Ras activation in 308, CH72-T3 or CC4A cell lines did not induce increased expression of ribosomal protein L11 (Figure 4A).

### Expression of activated Ras in Mdm2^C305F^ mutant MEFs induces a normal p53 response but does not up-regulate the expression of ribosomal proteins

While the data from mouse keratinocytes suggested that activated Ras may not induce ribosomal stress, the cell lines could not fully address the function of RP-Mdm2-p53 signaling in response to Ras activation. In order to investigate whether the Mdm2^C305F^ mutant protein, and thus decreased interaction between ribosomal proteins and Mdm2, could affect the p53 response to Ras activation, early passage *Mdm2^+/+^* and *Mdm2^C305F/C305F^* MEFs were stably infected with retroviruses encoding either H-Ras^G12V^ (a constitutively active form of H-Ras) or an empty vector control. Ras is known to induce cellular senescence via an intact p19Arf-Mdm2-p53 pathway in murine cells [25], [36]. Following infection with Ras virus, we observed comparable cell cycle arrest in *Mdm2^+/+^* and *Mdm2^C305F/C305F^* MEFs as evidenced by a similar decrease in cell number (Figure 4B). Ras expression was confirmed by western blot analysis and was comparable in *Mdm2^+/+^* and *Mdm2^C305F/C305F^* MEFs (Figure 4C).

To determine the effect of Ras expression on ribosomal protein levels, cell lysates were immunoblotted for expression of L5 and L11. Expression of activated Ras did not upregulate L11 or L5 in *Mdm2^C305F/C305F^* or *Mdm2^+/+^* MEFs (Figure 4C, lane 1 versus lane 2, lane 3 versus lane 4). To examine p53 response to Ras expression, cell lysates were also immunoblotted for p53. Ras induced p53 stabilization in both *Mdm2^C305F/C305F^* and *Mdm2^+/+^* MEFs (Figure 4C, lane1 versus lane2, lane 3 versus lane4). p53 was induced to a similar extent in *Mdm2^C305F/C305F^* and *Mdm2^+/+^* MEFs (Figure 4C, lane2 versus lane4), indicating that *Mdm2^C305F/C305F^* MEFs have a normal p53 response to Ras activation. These data suggest that Ras activation does not induce ribosomal stress in the cells tested, and that RP-Mdm2-p53 signaling may not be critical in response to Ras-induced oncogenic stress.

## Discussion

Recently several ribosomal proteins, including L11 [4], L5 [5] and L23 [6], [7] have been shown to bind Mdm2 at its zinc finger domain. Under conditions of ribosomal stress, free forms of ribosomal proteins are released into the nucleoplasm and bind to Mdm2, leading to p53 stabilization and activation [9]. Interestingly, a cancer-associated cysteine-to-phenylalanine point mutation in the zinc finger domain of Mdm2 disrupts binding of L11 and L5 to Mdm2 [10], and Mdm2^C305F^ mutant knock-in mice are deficient in p53 induction in response to induced ribosomal stress [11].

Additionally, the Mdm2 C305F mutation was recently shown to significantly accelerate B cell lymphomagenesis in an Eµ-Myc induced mouse model of B cell lymphoma [11], [45]. The ability of Myc to promote cell growth and proliferation is closely linked to its role in regulating ribosomal biogenesis, and in the case of Mdm2^C305F^ and Myc-induced lymphoma, ribosomal protein expression is elevated, however ribosomal proteins L11 and L5 are unable to bind and suppress Mdm2^C305F^, resulting in attenuation of p53 activation [11]. These findings established the RP-Mdm2-p53 pathway as a genuine barrier to Myc-induced tumorigenesis.

The current study examined whether the RP-Mdm2-p53 pathway acts as a general response to oncogenic stress by utilizing models of pRb inactivation and Ras activation. We now show that Mdm2 C305F mutation results in decreased prostate size and, unlike the situation in Myc-induced B cell lymphomagenesis [11], slows the progression of prostate tumorigenesis induced by inactivation of pRb family proteins in the well-characterized *APT_121_* mouse model of prostate cancer [32]. Immunohistochemical analysis showed a significant decrease in the percentage of ki67-positive cells in prostates isolated from *APT_121_;Mdm2^C305F/C305F^* versus *APT_121_;Mdm2^+/+^* mice, but no significant difference in TUNEL staining. These data suggest that the reduction in prostate size and slowed progression of prostate tumorigenesis induced by Mdm2 C305F mutation may be due to a defect in proliferation rather than an increase in cell death. Moreover, unlike the situation in Myc-induced lymphomagenesis in which ribosomal protein L11 expression was significantly increased [11], L11 expression was not induced by *APT_121_* (data not shown), suggesting that *APT_121_*-induced prostate cancer does not cause ribosomal stress. While *Mdm2^C305F/C305F^* mice exhibit smaller prostates than wild-type mice, the prostates from *Mdm2^C305F/C305F^* mice are normal in function and do not have developmental defects. p53 has recently been reported to promote cell survival through induction of *TIGAR* (*T*P53-*i*nduced *g*lycolysis and *a*poptosis *r*egulator) [46]. It is possible that disruption of RP-Mdm2-p53 signaling leads to a slightly lower level of p53 in the *Mdm2^C305F/C305F^* prostates. Under normal conditions, the slight difference in p53 level may not be critical for cell proliferation and growth, however, under oncogenic stress such as pRb inhitition, lower p53 levels may hinder cell proliferation in the *Mdm2^C305F/C305F^* prostates.

With regard to Ras activation, we show that constitutively active mutant Ras does not up-regulate the expression of ribosomal proteins either in mouse keratinocyte cell lines or when overexpressed in *Mdm2^+/+^* or *Mdm2^C305F/C305F^* MEFs. These data suggest that Ras activation does not induce ribosomal stress in the cells tested, and that RP-Mdm2-p53 signaling may not be critical in response to Ras-induced oncogenic stress.

While previous findings have shown that both RP-Mdm2 and p19Arf-Mdm2 signal to p53 and similarly suppress Myc-induced B cell lymphoma [11], [19], our data presented here suggest that disruption of RP-Mdm2 signaling does not accelerate *APT_121_*-induced prostate cancer. However, loss of p19Arf accelerates adenocarcinoma and stromal tumor development in *APT_121_*-induced prostate cancer, and isolated *APT_121_;p19Arf^−/−^* prostates exhibited a higher rate of proliferation and no significant difference in apoptosis compared with *APT_121_;p19Arf^+/+^* prostates. Thus, p19Arf-Mdm2-p53 signaling apparently inhibits *APT_121_*-induced prostate cancer progression by affecting cell proliferation. Furthermore, the phenotype observed in *APT_121_;p19Arf^−/−^* mice is consistent with that reported in a prior study on *APT_121_;p53^−/−^* mice [35], confirming the importance of p19Arf-Mdm2-p53 signaling in tumor suppression of *APT_121_*-induced prostate cancer.

In conclusion, the present study suggests that the p19Arf-Mdm2-p53 pathway suppresses *APT_121_*-induced prostate tumorigenesis. p19Arf-Mdm2-p53 may be a general pathway to suppress a wide range of oncogenic insults. However, p19Arf is not required for p53 response to ribosomal stress, while RP-Mdm2-p53 signaling is required [11]. The lack of ribosomal stress observed upon pRb inactivation and Ras activation also suggests that the PR-Mdm2-p53 pathway may not be a general barrier to oncogenic stress, but rather a specific response to ribosomal stress induced by oncogenes such as Myc. It is likely that p19Arf and RP are induced by different cellular conditions, oncogene stress and ribosomal stress respectively, both resulting in Mdm2 binding and activation of p53.

## Materials and Methods

### Ethics Statement

This study is approved by the Institutional Animal Care and Use Committee at the University of North Carolina at Chapel Hill. IACUC approval ID. 10–045.0. Mice were humanely euthanized by CO_2_ asphyxiation followed by a second method to ensure euthanasia. Mouse tumors and organs were fixed in formalin for histopathology and snap frozen for protein extraction.

### Mouse Breeding Strategies

Derivation of *APT_121_* (C57BL6/J;DBA2) transgenic mice was previously described [32]. To study the effect of the RP-Mdm2-p53 pathway on prostate tumorigenesis, *APT_121_* mice were mated to *Mdm2^C305F/C305F^* (C57BL6/J) mice that were generated and genotyped as previously described [11]. We used standard breeding strategies to produce *APT_121_;Mdm2^+/+^*, *APT_121_;Mdm2^C305F/C305F^* and nontransgenic male littermates *Mdm2^+/+^* and *Mdm2^C305F/C305F^* served as controls. To study the effect of the p19Arf-Mdm2-p53 pathway on prostate tumorigenesis, *APT_121_* mice were mated to *p19Arf^−/−^* (C57BL6/J; Sv129) mice that were generated and genotyped as previously described [25]. Mice harboring a homozygous deletion of *p19^ARF^* exon 1 were originally provided by C. J. Sherr and M. F. Roussel (St. Jude Children's Hospital) and maintained in Terry Van Dyke's lab (UNC-Chapel Hill). We used standard breeding strategies to produce *APT_121_;p19^ARF+/+^* and *APT_121_;p19^ARF−/−^* mice. Mice were bred and maintained under a protocol (10–045.0) approved by the Institutional Animal Care and Use Committee at the University of North Carolina Animal Care Facility. Mice were humanely euthanized by CO_2_ asphyxiation followed by a second method to ensure euthanasia. Mouse tumors and organs were fixed in formalin for histopathology and snap frozen for protein extraction.

### Measurement of prostate size

Prostate tissues from 6 month-old *APT_121;_Mdm2^+/+^* and *APT_121;_Mdm2^C305F/C305F^* mice as well as from their *Mdm2^+/+^* and *Mdm2^C305F/C305F^* littermate controls were excised, photographed, and weighed. All procedures involving mice were done according to a protocol approved by the University of North Carolina Institutional Animal Care and Use Committee.

### Histopathology

Prostate tissues from *APT_121;_Mdm2^+/+^* and *APT_121;_Mdm2^C305F/C305F^* mice as well as from their *Mdm2^+/+^* and *Mdm2^C305F/C305F^* non-tumorigenic littermate controls, were fixed overnight in 10%phosphate-buffered formalin and then transferred to 70%ethanol. Samples were sent to the UNC Histology Core Facility for paraffin embedding. Paraffin blocks were sectioned at 5-µm intervals for successive layers and stained with hematoxylin (Sigma-Aldrich, St. Louis, MO) and eosin for histopathology examination.

### Apoptosis analysis

Apoptosis levels of mouse prostate sections were assessed by the terminal deoxynucleotidyl transferase–mediated dUTP-biotin nick end labeling (TUNEL) assay (ApopTaq Peroxidease in situ Kit, Millipore, Temecula, CA). A ratio of TUNEL-positive stained cells to total cells counted was calculated. Statistical significance in differences in apoptosis levels between mice with different genotypes was evaluated by Student's *t* test (*P*<0.05 was considered significant).

### Proliferation analysis

Ki67 immunohistochemical staining of mouse prostate samples was used to detect proliferating cells. Antigen retrieval for antibody on formalin-fixed paraffin sections was done by boiling paraffin samples in citrate buffer (pH 6.0) for 15 min. Endogenous peroxidase activity was quenched by incubation in 3%H_2_O_2_ in methanol for 10 minutes. Antibody detection was done by using purified mouse anti-human Ki67 primary antibody (BD Pharmigen, San Diego, CA) and biotin-conjugated anti-mouse secondary antibody (Vector Laboratories, Burlingame, CA). An avidin-biotin-peroxidase kit (Vectastain Elite, Vector Laboratories) with diaminobenzidine was used as a chromogen. A ratio of positive stained cells to total cells was calculated. Statistical significance in differences in proliferation levels between mice with different genotypes was evaluated by Student's *t* test (*P*<0.05 was considered significant).

### Culture of cells

Mouse keratinocyte cell lines and low-calcium culture media were provided by Dr. Marcelo Rodriguez-Puebla at North Carolina State University. MEF cells were isolated on embryonic day 13.5 (E 13.5) and cultured in DMEM (GIBCO, Carlsbad, CA) supplemented with 10%fetal bovine serum (GIBCO) and penicillin-streptomycin (GIBCO).

### Retroviral infection of MEF cells

293 QBT cells [47] were transfected with plasmids: pVPack-Eco (viral coat protein plasmid for infecting mouse and rat cells), pVPack-Gag-Pol (viral protein plasmid) and pBabe or pBabe-H-Ras12V. Fugene HD kit was utilized for transfections, following manufacturer's instructions (Roche diagnostics, Indianapolis, IN). Virus-containing medium from 293 QBT cells was filtered through a 0.45 µm syringe-tip filter and mixed with fresh medium at the ratio of 1∶1. Polybrene was added to the mixed medium to a final concentration of 6 µg/ml. The medium from primary mouse embryo fibroblasts (MEF) cells was removed and replaced with virus-containing medium. MEF cells were infected with a retrovirus derived from pBabe-puro-H-Ras12V or empty vector as a control, and stable polyclonal populations were selected by puromycin resistance.

### Protein detection

Prostate tissues from *APT_121;_Mdm2^+/+^* and *APT_121;_Mdm2^C305F/C305F^* as well as from their *Mdm2^+/+^* and *Mdm2^C305F/C305F^* non-tumorigenic littermate controls were homogenized on ice and lysed in 0.5%NP-40 lysis buffer. Cultured cells (mouse keratinocytes and MEFs) were also lysed in 0.5%NP-40 lysis buffer. Total cellular lysates were run on a 12.5%SDS-polyacrymide gel followed by immunoblotting using standard procedures. Mouse monoclonal anti-p53 (NCL-505, Novocastra Laboratories, Newcastle upon Tyne, England) and anti-actin (MAB1501, Chemicon International, Temecula, CA) antibodies were purchased commercially. Rabbit polyclonal antibodies to L5 and L11 were produced as previously described [10].
